# Supplementary material for: The real-world outcomes of multiple myeloma patients treated with daratumumab
Source: PLoS One. 2021 Oct 13;16(10):e0258487. doi: 10.1371/journal.pone.0258487 (PMC8513840; doi:10.1371/journal.pone.0258487)
Supplement: S1 File — (PDF) [file pone.0258487.s001.pdf]

1

Supplementary material.

The real-world outcomes of multiple myeloma patients treated with daratumumab as monotherapy, in combination with immunomodulatory drugs and proteasome inhibitors by Szabo et al. Corresponding author AJV.

Agoston Gyula Szabo, Tobias Wirenfeldt Klausen, Mette Bøegh Levring, Birgitte Preiss, Carsten Helleberg, Marie Fredslund Breinholt, Niels Emil-Hermansen, Lise Mette Rahbek Gjerdrum, Søren Thorgaard Bønløkke, Katrine Nielsen, Eigil Kjeldsen, Katrine Fladeland Iversen, Elena Manuela Teodorescu, Marveh Dokhi, Eva Kurt; Casper Strandholdt, Mette Klarskov Andersen, Annette Juul Vangsted

Corresponding author:

Associate professor Annette Juul Vangsted

Senior Consultant, DMSc

Copenhagen University Hospital, Rigshospitalet

Department of Hematology, Blegdamsvej 9, DK-2100 Copenhagen

Tel no: +45 35451452

e-mail: annette.juul.vangsted@regionh.dk

The real-world outcomes of multiple myeloma patients treated with daratumumab as monotherapy, in combination with immunomodulatory drugs and proteasome inhibitors by Szabo et al. Corresponding author AJV.

Supplementary table 1. Available treatment options in Denmark.

	HDM-ASCT-eligible	HDM-ASCT-ineligible
1. line or later	CVD + HDM-ASCT	RD
	VTD + HDM-ASCT	MPV
	VRD[€] + HDM-ASCT	VRD[€]
	R-maintenance[#]	MP
		BP
		CP
2. line and later	KD[&]	
	PVD[&]	
	KRD[*]	
	ERD[*]	
	IRD[@]	
	DRD[§]	
	DVD[§]	
	TD	
	VD	
3. line and later	PD	
	D-mono[*]	

&: Approved 1. July 2016; *: Approved 6. September 2016; @: Approved 1. March 2017; §: Approved 19. July 2017; €: Approved 15. May 2019; : Approved 11. October 2019

Abbreviations: HDM-ASCT=high-dose melphalan followed by autologous stem cell transplantation; CVD=cyclophosphamide-bortezomib-dexamethasone, VTD=bortezomib-thalidomide-dexamethasone; VRD=bortezomib-lenalidomide-dexamethasone; R=lenalidomide; RD=lenalidomide-dexamethasone; MPV=melphalan-prednisolone-bortezomib; BP=bendamustine-prednisolone; CP=cyclophosphamide-prednisolone; KD=carfilzomib-dexamethasone; PVD=panobinostat-bortezomib-dexamethasone; KRD=carfilzomib-lenalidomide-dexamethasone; ERD=elotuzumab-lenalidomide-dexamethasone; IRD=ixazomib-lenalidomide-dexamethasone; DRD=daratumumab-lenalidomide-dexamethasone; DVD=daratumumab-bortezomib-dexamethasone; TD=thalidomide-dexamethasone; VD=bortezomib-dexamethasone; PD=pomalidomide-dexamethasone; D-mono=daratumumab monotherapy

The real-world outcomes of multiple myeloma patients treated with daratumumab as monotherapy, in combination with immunomodulatory drugs and proteasome inhibitors by Szabo et al. Corresponding author AJV.

Supplementary table 2. Data collected in the study

	At diagnosis from the DMMR	During follow-up
Patient characteristics	Sex, gender, age, date of diagnosis, date of death,	date of last contact
Myeloma characteristics	B2M, albumin, creatinine, LDH, ISS, M-protein	
FISH	Del17p, t(4,14), t(14,16) amp1 q	Del17p, t(4,14), t(14,16) amp1 q
Treatment before and after daratumumab	First line treatment	Bortezomib, carfilzomib, ixazomib, thalidomide, lenalidomide, pomalidomide, cyclophosphamide, melphalan high and low dose, doxorubicine, liposomal doxorubicin, melflufen, bendamustine, daratumumab, elotuzumab, panobinostat, vorinostat, vidaza, prednisone, dexamethasone and other
Reasons for stopping treatment		Progression, toxicity, poor PS, insufficient response, acceptable treatment effect/plateau phase, planned according to protocol, patients wish, death of the patients and other
Toxicity		Haematological, cardiac arrythmia and ischemia, diarrhoea, other gastrointestinal problems, hepatic toxicity, immune dysfunction and infections, skeletal-muscular symptom, neuropathy, other neurological disorders, psychiatric diseases, airway problems, dermatological problems including rash, thrombosis, other vascular problems and other
Response to treatment		Paraclinical sCR and CR, VGPR, PR, MR, SD and progression according to IMW criteria

DMMR: Danish multiple myeloma registry

The real-world outcomes of multiple myeloma patients treated with daratumumab as monotherapy, in combination with immunomodulatory drugs and proteasome inhibitors by Szabo et al. Corresponding author AJV.

Supplementary table 3. Cytogenetic abnormalities in the study population assessed by fluorescence in situ hybridization

	Cut-off	N (%)	Missing N (%)	Positive N (%)	Positive N (%) in patients with TNT on Da	Positive N (%) in patients still on Da	P value
t(4:14)	10%	467 (73.5)	168 (26.5)	61 (13.9%)	42 (13.8)	19 (11.7)	0.07
t(14:16)	10%	458 (72.1)	177 (27.9)	19 (4.1%)	16 (5.4)	3 (1.9)	0.53
Del17p	10%	485 (76.4)	150 (23.6)	66 (13.6%)	49 (15.6)	17 (10.0)	0.09
Amp1q	10%	468 (73.7)	167 (26.3)	138 (29.5%)	96 (31.5)	42 (25.8)	0.21
t(4;14) t(14:16) del17p	10%	463 (72.9)	172 (27.1)	135 (29.2)	97 (32.2)	38 (23.5)	0.048

Abbreviations: Da=the first daratumumab-containing line of therapy; TNT=time to next treatment, N=number; %=percentage.

The real-world outcomes of multiple myeloma patients treated with daratumumab as monotherapy, in combination with immunomodulatory drugs and proteasome inhibitors by Szabo et al. Corresponding author AJV.

Supplementary table 4. Number of lines of therapy in the study population throughout the entire course of their disease

All patients

no of lines	n	%	cumsum	cum %
22	1	0,2	1	0,2%
18	3	0,5	4	0,6%
17	1	0,2	5	0,8%
16	6	0,9	11	1,7%
15	1	0,2	12	1,9%
14	1	0,2	13	2,0%
13	10	1,6	23	3,6%
12	12	1,9	35	5,5%
11	15	2,4	50	7,9%
10	16	2,5	66	10,4%
9	30	4,7	96	15,1%
8	41	6,5	137	21,6%
7	39	6,1	176	27,7%
6	61	9,6	237	37,3%
5	88	13,9	325	51,2%
4	92	14,5	417	65,7%
3	113	17,8	530	83,5%
2	99	15,6	629	99,1%
1	6	0,9	635	100,0%

Patients treated with HDT

no of lines	n	%	cumsum	cum %
18	2	0,6	2	0,6%
17	1	0,3	3	1,0%
16	2	0,6	5	1,6%
14	1	0,3	6	1,9%
13	7	2,3	13	4,2%
12	8	2,6	21	6,8%
11	5	1,6	26	8,4%
10	9	2,9	35	11,3%
9	12	3,9	47	15,2%
8	21	6,8	68	22,0%

7	25	8,1	93	30,1%
6	32	10,4	125	40,5%
5	48	15,5	173	56,0%
4	42	13,6	215	69,6%
3	49	15,9	264	85,4%
2	44	14,2	308	99,7%
1	1	0,3	309	100,0%

Non-HDT patients

no of lines	n	%	cumsum	cum %
22	1	0,3	1	0,3%
18	1	0,3	2	0,6%
16	4	1,2	6	1,8%
15	1	0,3	7	2,1%
13	3	0,9	10	3,1%
12	4	1,2	14	4,3%
11	10	3,1	24	7,4%
10	7	2,1	31	9,5%
9	18	5,5	49	15,0%
8	20	6,1	69	21,2%
7	14	4,3	83	25,5%
6	29	8,9	112	34,4%
5	40	12,3	152	46,6%
4	50	15,3	202	62,0%
3	64	19,6	266	81,6%
2	55	16,9	321	98,5%
1	5	1,5	326	100,0%

HDT: high-dose melphalan with hematopoietic stem cell support

The real-world outcomes of multiple myeloma patients treated with daratumumab as monotherapy, in combination with immunomodulatory drugs and proteasome inhibitors by Szabo et al. Corresponding author AJV.

Supplementary table 5. Characteristics of 635 patients treated with daratumumab

N= 635	Da-mono	Da-IMiD	Da-PI	Da-other	p value[1]	Patients still on Da
N (%)	176 (27.7)	364 (57.3)	71 (11.2)	24 (3.8)		225 (35.4)
Diagnose before 2009; N (%)	29 (16.5)	36 (9.9)	9 (12.7)	3 (12.5)	0.088	19 (8.4)
Diagnose 2009-2019; N (%)	147 (85.4)	328 (90.1)	62 (83.3)	21 (87.5)	0.071	206 (91.6)
Age at diagnosis; median (IQR)	67 (60-72)	65 (57-71)	65 (55-71)	57 (50-67)	0.024	66 (58-71)
Age at start of treatment; median IQR	72 (67-77)	70 (63-74)	70 (62-75)	63 (51-74)	0.002	70 (62-75)
Gender male/female; (% male)	105/71 (59.7)	201/163 (55.2)	37/34 (52.1)	14/10 (41.7)	0.48	120/105 (53.3)
ISS					0.64	
I; no (%)	46 (32.6)	91 (29.4)	16 (26.2)	9 (45.0)		54 (28.9)
II; no (%)	53 (37.6)	119 (38.5)	27 (44.3)	4 (20.0)		81 (43.3)
III; no (%)	42 (29.8)	99 (32.0)	18 (29.5)	7 (35.0)		52 (27.8)
Missing N	35	55	10	4 (16.7)		38
M-protein type					0.24	
IgG ; N (%)	100 (60.6)	187 (57.4)	43 (67.2)	7 (36.8)		124 (61.1)
IgA ; N (%)	33 (20.0)	77 (23.6)	14 (21.9)	7 (36.8)		39 (19.2)
Light chain only; N (%)	26 (15.7)	50 (15.3)	7 (10.9)	5 (26.3)		33 (13.3)
Other; N (%)	6 (3.6)	12 (3.7)	0	0		7 (3.4)
Missing; N	11	38	7	5		22
LDH elevated at diagnosis; N (%) [N missing]	30 (18.8) [16]	106 (31.9) [32]	21 (31.8) [5]	5 (23.8) [3]	0.020	64 (26.2)
Creatinine above 177µmol/L; N (%) [N missing]	27 (16.0) [7]	49 (14.5) [25]	9 (13.6) [5]	5 (22.7) [2]	0.77	30 (14.2)
High-risk CA: at least one of del17p, t(14;16), t(4;14);N (%) [N missing]	31 (24.8) [51]	81 (30.1) [95]	18 (36.0) [21]	5 (26.9)	0.30	19 (11.7)
Amp1q; no (%) [N missing]	40 (32.0) [51]	79 (28.9) [91]	14 (27.5) [20]	5(26.9)	0.77	3 (1.9)
Prior treatment before daratumumab						
HDM-ASCT in first line	59 (33.5)	190 (52.2)	41 (57.7)	19 (79.2)	<0.001	107 (47.6)
Relapse < 18 months from HDT	15 (8.5)	68 (18.7)	12 (16.9)	10 (41.7)	0.29	34 (15.1)
Bortezomib; N (%)	166 (94.3)	340**(96.0)	66 (93.0)	24 (100)	0.44	206 ** (93.2)
Lenalidomide; N (%)	157 (89.2)	158** (44.6)	56 (78.9)	18 (75.0)	<0.0001	93 ** (42.1)
Carfilzomib; N (%)	57 (32.4)	72** (20.3)	14 (19.7)	6 (25.0)	0.006	16 **(7.2)
Pomalidomide; N (%)	78 (44.3)	35** (9.9)	16 (22.5)	7 (29.2)	<0.0001	17 ** (7.7)
IMiDs and PI; N (%)	158 (89.8)	159** (44.9)	55 (77.5)	19 (79.2)	<0.0001	92 **(41.6)
Quadruple-exposed; N (%)	32 (18.2)	13** (3.7)	5 (7.0)	3 (12.5)	<0.0001	1** (0.5)
Number of lines of therapy given before daratumumab; median (range)	5 (2-16)	3 (1-11)	4 (2-10)	2 (1-3)	<0.0001	
Number of lines of therapy given before daratumumab						
	0	10 (2.7)	0	0		4 (1.8)

1.	8 (4.5)	126 (35.6)	9 (12.7)	6 (25.0)		87 (38.7)
2.	27 (15.3)	88 (24.9)	21 (29.6)	7 (29.2)		57 (25.3)
3.	46 (26.1)	61 (17.2)	9 (12.7)	6 (25.0)		43 (19.1)
4 or more	95 (54.0)	79 (22.3)	32 (35.0)	5 (20.8)		34 (15.1)
Time from diagnosis to start of daratumumab; mo; median (IQR)	48.2 (26.8-79.8)	37.9 (18.1-69.4)	49.2 (30.2-91.1)	28.5 (7.7-86.7)	0.002	38.9 (17.9-71.5)
Median follow-up after start of daratumumab; mo	22.7	17.8	20.3	27.7		15.9

N=number; IQR: interquartile range; mo: months; LDH upper reference 205 U/L. .Quadruple-exposed=previously treated with both bortezomib, lenalidomide, pomalidomide and carfilzomib. ** without the 10 patients that received daratumumab at first line.. [1]The p-value describes the difference between Da-IMiDs, Da-PI, and Da-mono

The real-world outcomes of multiple myeloma patients treated with daratumumab as monotherapy, in combination with immunomodulatory drugs and proteasome inhibitors by Szabo et al. Corresponding author AJV.

Supplementary table 6. Overall response and time to next treatment in patients treated with daratumumab regimens

N=635	**Da-mono**<br>**N=176**	**Da-IMiD**<br>**N=364**	**Da-PI**<br>**N=71**	**Dara-other**<br>**N=24**	**p value[1]**
**Outcome all**					
ORR, N (%)	79 (44.9)	293 (80.5)	43 (60.6)	13 (54.2)	<0.0001
≥VGPR, N (%)	34 (19.3)	204 (56.0)	23 (32.4)	6 (25.0)	<0.0001
TNT all lines, median mo (CI)[N]	4.9 (3.7-5.8)[176]	16.1 (13.7-20.3)[364]	5.3 (3.5-8.2)[71]	5.6 (2.8-12.7)[24]	<0.0001
TNT 2. line, median mo (CI)[N]	5.0 (2.8-NR)[8]	25.9 (17.8-NR) [126]	NR (3.0-NR) [9]	4.9 (1.8-NR)[6]	
TNT 3. line, median mo (CI)[N]	5.5 (2.6-NR) [27]	18.8 (13.2-NR) [88]	5.5 (3.5-11.4) [21]	2.7(1.4-NR)[7]	
TNT 4. line, median mo (CI)[N]	9.2 (5.6-18.2) [46]	15.0 (10.2-NR) [61]	3.8 (2.5-NR) [9]	7.5(6.4-NR)[6]	
TNT 5. line, median mo (CI)[N]	2.8 (1.8-6.0) [28]	10.7 (5.4-NR) [35]	8.6 (2.4-NR) [8]	NR)[1]	
TNT ≥ 6. line, median mo (CI)[N]	3.9 (2.7-5.5) [67]	6.4 (5.1-10.3)[44]	3.7 (3.9-7.3)[24]	3.4 (0.8-NR)[4]	
**Outcome for high-risk (del17p, t(14;16), t(4;14);**				N= 5[2]	
ORR, N (%)	10 (32.3)	59 (72.8)	8 (44.4)	[2]	0.0002
≥VGPR, N (%)	4 (12.9)	42 (51.9)	4 (22.2)	[2]	0.0002
TNT, median mo (CI)[N]	3.7 (1.9-6.4)[31]	11.8 (7.3-14.6)[81]	4.0 (2.5-10.7)[18]	[2]	0.002
**Outcome for amp1q**				N=5[2]	
OOR, N (%)	17 (42.5)	60 (75.9)	7 (50.0)	[2]	0.0009
≥VGPR, N (%)	8 (20.0)	44 (55.7)	5 (35.7)	[2]	0.0008
TNT, median mo (CI)[N]	3.4(2.3-5.6)[40]	14.7 (12.4-25.8)[79]	3.4 (2.5-NR)[14]	[2]	<0.0001

Mo =months, N=number; CI= confidence interval; NR=not reached. ** without the 10 patients that received daratumumab at first line, Other=non-secretory, IgE and IgM.

[1]The p-value describes the difference between Da-IMiDs, Da-PI, and Da-mono.

[2]The number are too small to be presented. Dara-other include many different treatment strategies such as IMiD-PI combinations, cyclophosphamide, bendamustine, panobinostat, doxorubicin or melphalan

The real-world outcomes of multiple myeloma patients treated with daratumumab as monotherapy, in combination with immunomodulatory drugs and proteasome inhibitors by Szabo et al. Corresponding author AJV.

Supplementary table 7. Response to daratumumab regimens according to timing in line of therapy

Response N= 635	Da-mono N (%)	Da-IMiD N (%)	Da-PI N (%)	Dara-other N(%)	P value[1]	225 without TNT N (%)
Response All lines	N=176	N=364	N=71	N= 24		
≥VGPR	34 (19.3)	204 (56.0)	23 (32.4)	6 (25)		165 (73.3%)
PR	45 (25.6)	89 (24.5)	20 (28.2)	7(29.2)		43 (19.1)
≤ MR	97 (55.1)	71 (19.5)	28 (39.4)	11(45.8)		17 (7.6)
total	176	364	71		<0.0001	225
Response 2. line				2		
≥VGPR	2 (25.5)	89 (70.6)	5 (55.6)			71 (81.6)
PR	1 (12.5)	19 (15.1)	3 (33.3)			9 (10.3)
≤ MR	5 (62.5)	18 (14.3)	1 (11.1)			7 (8.0)
total	8	126	9		0.008	87
Response 3. line				2		
≥VGPR	5 (18.5)	48 (54.5)	9 (42.9)			43 (75.4)
PR	9 (33.3)	26 (29.5)	6 (28.6)			13 (22.8)
≤ MR	13 (48.1)	14 (15.9)	6 (28.6)			1 (1.8)
total	27	88	21		0.0007	57
Response 4. line				2		
≥VGPR	14 (30.4)	33 (54.1)	1 (11.1)			30 (69.8)
PR	10 (21.7)	13 (21.3)	2 (22.2)			10 (23.3)
≤ MR	22 (47.8)	15 (24.6)	6 (54.0)			3 (7.0)
total	46	61	9		0.003	43
Response 5. line						
≥VGPR	2 (7.1)	12 (34.3)	4 (50.0)			10 (52.6)
PR	7 (25.0)	11 (31.4)	2 (25.0)			6 (31.6)
≤ MR	19 (67.9)	12 (34.3)	2 (25.0)			3 (15.9)
total	28	35	8		0.004	19
Response ≥ 6. line				1		
≥VGPR	11 (16.4)	16 (36.4)	4 (16.7)			9 (60.0)
PR	18 (26.9)	16 (36.4)	7 (29.2)			3 (20.0)
≤ MR	38 (56.7)	12 (27.3)	13 (54.2)			3 (20.0)
total	67	44	24		0.004	15

Supplementary table 8. Number of quadruple-exposed patients prior to their first daratumumab-containing line of therapy

Line of therapy for Da	Quadruple-exposed before Da (bortezomib, carfilzomib, lenalidomide and pomalidomide), no of patients	Not quadruple-exposed before Da no of patients
2	0	149
3	0	143
4	4	118
5	6	66
6+	43	96

Fifty-three patients were quadruple-exposed (bortezomib, carfilzomib, lenalidomide and pomalidomide) before first treatment with daratumumab (Da). TNT for Da in quadruple-exposed patients was 3,7 months versus 11.0 months for other patients (p <0.0001).

Supplementary table 9. Combinations and timing of the first daratumumab-containing line of therapy

Line of therapy for Da	Da-mono, N (%)	Da-IMiD; N(%)	Da-PI, N (%)	Da-other, N (%)
1	0	10 (1.6)	0	0
2	8 (1.3)	126 (19.8)	9 (1.4)	6 (0.9)
3	27 (4.3)	88 (13.6)	21 (3.3)	7 (1.1)
4	46 (7.2)	61 (9.6)	9 (1.4)	6 (0.9)
5	28 (4.4)	35 (5.5)	8 (1.3)	1 (0.15)
6 and more	67 (10.9)	44 (6.9)	24 (3.8)	4 (0.6)

Formateret tabel

Abbreviations: Da=the first daratumumab-containing line of therapy; Da-mono=daratumumab monotherapy; Da-IMiD=daratumumab in combination with an immunomodulatory agent; Da-PI=daratumumab in combination with a proteasome inhibitor; Da-other= daratumumab in combination with with IMiD-PI combinations, cyclophosphamide, bendamustine, panobinostat, doxorubicin or melphalan N=number.

In patients treated with Da-IMiD, the IMiD partner was lenalidomide in 317 patients (87.1%) and pomalidomide in 47 (12.9%) patients. In Da-PI, the PI partner was bortezomib in 70 patients and ixazomib in 1 patient. Daratumumab was used in other combinations in 24 patients (3.8%). Ten patients (1.6%) received Da as first-line treatment (in the MAIA study), 149 patients (23.5%) as 2. line, 143 patients (22.5%) as 3. line, 122 patients (19.2%) as 4. line, 72 patients (11.3%) as 5. line and 135 patients (21.9%) as a 6. or later LOT. At data cutoff, 225 (35.4%) patients were still on Da; of which 4 patients received Da as 1. line, 87 patients as 2. line, 57 patients as 3. line, 43 patients as 4. line, 19 patients as 5. line and 15 patients as 6. or later LOT. Of the 225 patients who were still on Da at data cutoff, 31 patients were treated with Da-mono, 178 patients were treated with Da-IMiD, 12 patients were treated with Da-PI and 4 patients treated with Da in other combinations.

Supplementary table 10. TNT for patients with high-risk cytogenetic abnormalities

	N	TNT in month	CI in month	HR (95% CI)	P value
Standard risk	247	11.7	9.5-15.6	1	
Amp1q	75	9.8	6.5-16.9	1.08 (0.78-1.49)	0.65
High-risk	77	7.6	5.6-11.9	1.35 (0.98-1.84)	0.066
High-risk + amp1q	55	5.6	3.6-12.4	1.68 (1.19-2.36)	0.003
Missing	181	10.4	7.3-14.5	1.05 (0.83-1.34)	0.68
Amp1q	75	9.8	6.5-16.9	1	
High-risk + amp1q	55	5.6	3.6-12.4	1.56 (1.03-2.35)	0.036

N=number; HR=hazard ratio; CI=confidence interval; TNT=time to next treatment

Supplementary table 11. TNT for patients with and without t(4;14), t(14;16), del(17p) according to timing

High-risk CA	TNT 2. line	TNT 3. line	TNT 4. line	TNT 5.	TNT ≥6 lines
	mo (CI), [N]	mo (CI), [N]	mo (CI), [N]	mo (CI), [N]	mo, (CI), [N]
Not present	25.9 (17.8-NR) [87]	13.2 (8.8-20.1) [74]	11.3 (9.8-20.0) [60]	6.0 (2.4-12.0) [39]	4.3 (2.9-6.2) [61]
Present	11.8 (7.7-NR) [33]	6.6 (2.9-7.6) [33]	4.3 (3.5-NR) [25]	5.5 (2.4-NR) [15]	4.2 (2.3-13.1) [25]

High-risk cytogenetic abnormalities (CA): t(4;14), t(14;16) and del(17p); N=number; mo=months; CI=confidence interval; TNT=time to next treatment

Supplementary table 12. TNT for daratumumab, irrespectively of combination, according to line of therapy compared to therapy not including daratumumab

Line of therapy for Da	N	TNT in months for Da	No of patients	TNT in months without Da	P value	HR (CI)
2	148	25.9 (15.6-NR)	476	11.1 (9.7-NR)	<0.0001	0.52 (0.40-0.68)
3	143	11.4 (7.4-17.6)	331	8.8 (7.9-9.9)	0.012	0.74 (0.58-0.94)
4	122	11.1 (6.7-16.5)	211	7.7 (6.7-9.7)	0.0005	0.63 (0.49-0.82)
5	79	5.5 (2.9-12.3)	139	6.4 (4.9-8.7)	0.34	0.85 (0.62-1.18)
≥6	240	3.9 (3.5-4.8)	362	4.5 (3.9-5.2)	0.93	1.01 (0.85-1.20)

Da=the first daratumumab-containing line of therapy N=number; CI=confidence interval; TNT=time to next treatment; HR=hazard ratio;

Supplementary table 13. Univariate and multivariate analysis of factors affecting time to next treatment.

	Univariate		Multivariate	
	HR (95% CI)	p	HR (95% CI)	p
Line of therapy of first daratumumab exposure**		<0.0001*		<0.0001*
2 (reference)	1 (reference)		1 (reference)	
3	1.69 (1.22-2.34)	0.002	1.86 (1.26-2.76)	0.002
4	1.73 (1.24-2.41)	0.001	1.59 (1.03-2.47)	0.037
5	2.51 (1.74-3.62)	<0.0001	2.30 (1.46-3.62)	0.0003
6+	3.61 (2.65-4.90)	<0.0001	3.13 (2.05-4.79)	<0.0001
Regimen**		<0.0001*		<0.0001*
Da-IMiD (reference)	1 (ref)		1 (ref)	
Da-mono	2.41 (1.93-3.00)	<0.0001	1.90 (1.41-2.58)	<0.0001
Da-PI	2.35 (1.75-3.15)	<0.0001	2.13 (1.46-3.62)	<0.0001
Other	2.38 (1.50-3.79)	0.0002	2.56 (1.42-4.60)	0.002
HDT 1st line	1.01 (0.83-1.23)	0.92	-	
Age	1.01 (0.99-1.02)	0.34	-	
M-protein isotype: IgA	1.29 (1.02-1.65)	0.037	1.26 (0.94-1.68)	0.12
High-risk CA	1.42 (1.11-1.82)	0.005	1.30 (1.00-1.69)	0.052
Amp1q	1.18 (0.92-1.51)	0.19	-	

*) p for all variables, ** excluding patients treated with daratumumab in first line

Supplementary table 14. Causes of discontinuation of the first daratumumab-containing line of therapy

Cause of discontinuation*	All	Da-mono	Da-IMiD	Da-PI
progression	245 (59.8; 38.6)	87 (60.0; 49.4)	107 (57.5; 29.4)	38 (63.3; 53.5)
toxicity	56 (13.7; 8.8)	6 (4.1; 3.4)	35 (18.8; 9.6)	11 (18.3; 15.5)
insufficient response	49 (12.0; 7.7)	37 (25.5; 21.0)	9 (4.8; 2.5)	1 (1.7; 1.4)
patient wish	8 (2.0; 1.3)	2 (1.4; 1.1)	6 (3.2; 1.6)	0
poor performance status	10 (2.4; 1.6)	3 (2.1; 1.7)	3(1.6; 0.8)	4 (6.7; 5.6)
death	25 (6.1; 3.9)	5 (3.4; 2.8)	17 (9.1; 4.7)	3 (5.0; 4.2)
other reasons	4 (1.0; 0.6)	1 (0.7; 0.6)	2 (1.1; 0.5)	1 (1.7; 1.4)
missing information	13 (3.2; 2.0)	4 (2.8; 2.3)	7 (3.8; 1.9)	2 (3.3; 2.8)
Total	410	145	186	59

*: n (% of discontinuations in group; % of all patients treated in group)

Abbreviations: Da-mono=daratumumab monotherapy; Da-IMiD=daratumumab in combination with an immunomodulatory agent; Da-PI=daratumumab in combination with a proteasome inhibitor; n=number; other reasons=2 cases of myelodysplastic syndrome, 1 case of disseminated adenocarcinoma with thrombotic microangiopathy, 1 case of volvulus requiring acute surgery.

All patient in this study were treated with subcutaneous bortezomib when treated with daratumumab, which has been the standard of care in Denmark since 2011.

The real-world outcomes of multiple myeloma patients treated with daratumumab as monotherapy, in combination with immunomodulatory drugs and proteasome inhibitors by Szabo et al. Corresponding author AJV.

Supplementary table 15. Reasons for discontinuation of the first daratumumab-containing line of therapy

Toxicity to daratumumab	All patients N (%) (N 56)	% of all treated (N 635)	Da-Mono No (%) (N 176)	Da-IMiDs No (%) (N 364)	Da-PI No (%) (N 71)
Infection	17 (30.4)	2.7	3 (1.7)	10 (2.7)	2 (2.8)
Neuropathy	9 (16.1)	1.4	1 (0.6)	4 (1.4)	4 (5.6)
Marrow suppression	6 (10.7)	0.9	2 (1.1)	1 (0.2)	2 (2.8)
GI symptoms	6 (10.7)	0.9		4 (1.1)	2 (2.8)
Cardiovascular	4 (7.1)	0.6		4 (1.1)	
Thrombosis	4 (7.1)	0.6		4 (1.1)	
Musculoskeletal	4 (7.1)	0.6		4 (1.1)	
Psychiatric reasons	4 (7.1)	0.6	1 (0.6)	2 (0.5)	1 (1.4)
Fatique	5 (8.9)	0.8		4 (1.1)	
Kidney impairement	4 (7.1)	0.6		2 (0.5)	1 (1.4)
Infusion reactions	5 (8.9)	0.8	1 (0.6)	4 (1.1)	
Other	11 (19.6)	1.7	1 (0.6)	8 (2.2)	2 (2.8)

Other: avascular necrosis, leukocytoclastic vasculitis, dyspnoea, skin changes, neurological causes, headache, relapse of other cancer, unknown respiratory problems, arteritis temporalis.

Abbreviations: 1Da=the first daratumumab-containing line of therapy; Da-mono=daratumumab monotherapy; Da-IMiD=daratumumab in combination with an immunomodulatory agent; Da-PI=daratumumab in combination with a proteasome inhibitor; n=number; %=percentage; Supplementary Table 15 is to be viewed in relation to Supplementary Table 14. As shown in Supplementary Table 14, toxicity (in general) was the cause of discontinuation of the first daratumumab-containing line of therapy in 56 cases, which was 13.7% of all discontinuations. Supplementary Table 15 shows the type of toxicity in the 56 cases. Neuropathy was reported as the toxicity leading to discontinuation in 9 cases in total, across all daratumumab regimens, representing 16.1% of discontinuations due to toxicity. In total, this patient number represented 1.4% of the entire cohort of daratumumab-treated patients. The distribution of the 9 cases of neuropathy across the daratumumab regimens is shown in this table: 1 patient in Da-mono, 4 patients in Da-IMiD, 4 patients in Da-PI.

Supplementary figure 1. Strategy for time to next treatment

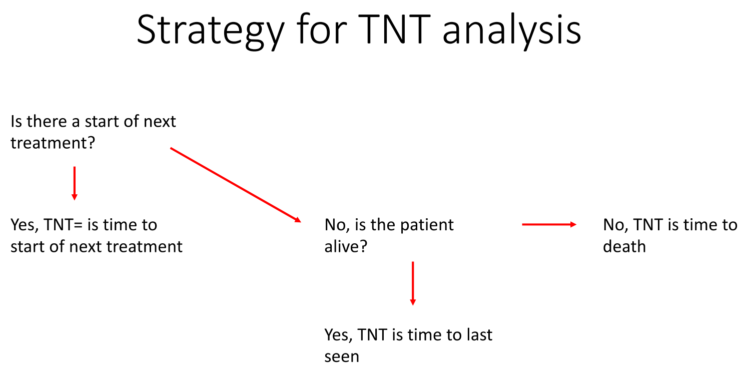

TNT= time to next treatment

Supplementary figure 2. Timing of the first daratumumab-containing line of therapy

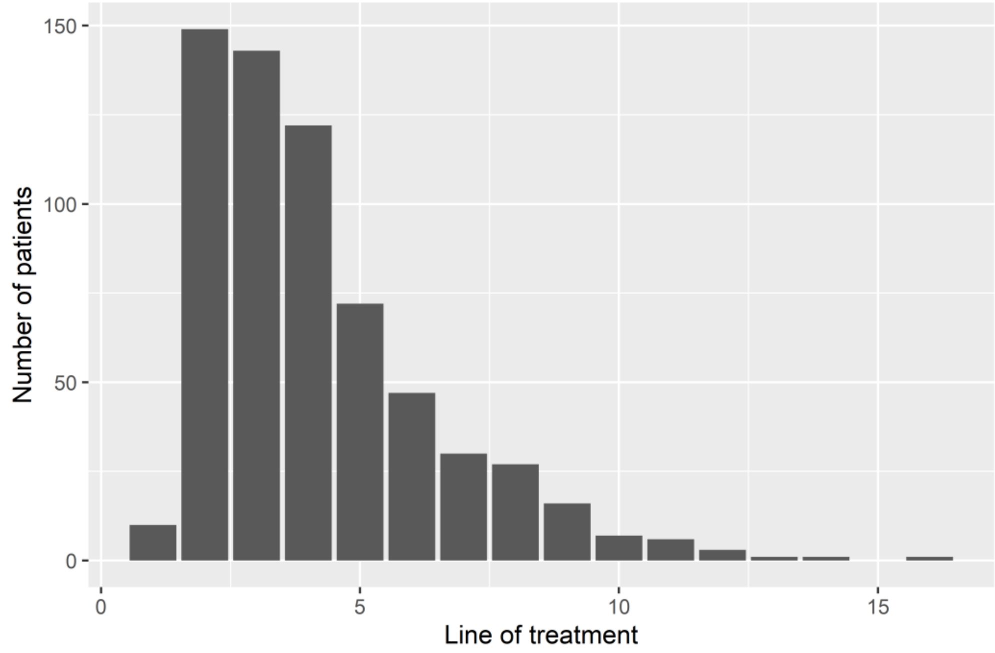

Line	1	2	3	4	5	6	7	8	9	10	11	12	13	14		16
N	10	149	143	122	72	47	30	27	16	7	6	3	1	1		1

Legends to figure: The X-axis presents in which line of treatment daratumumab was given for the first time. The Y-axis show the number of patients.

Supplementary figure 3. Response to first daratumumab-containing line of therapy depending on regimens

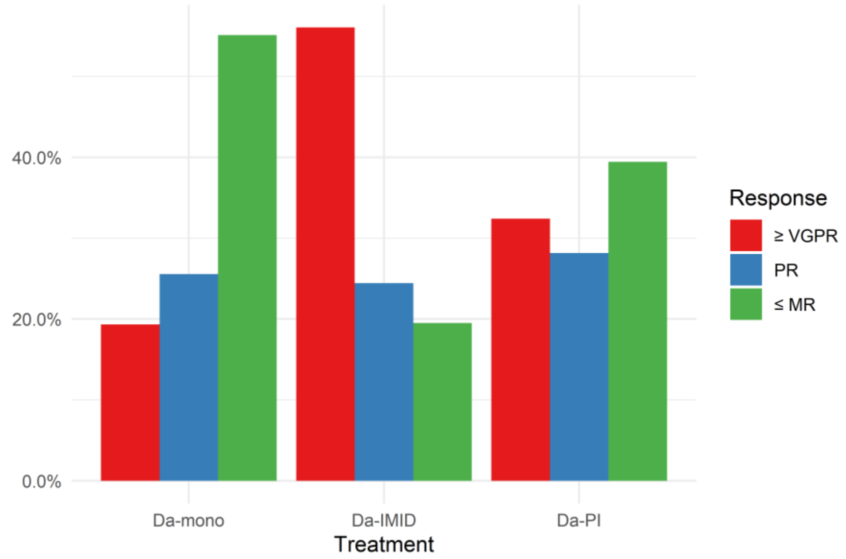

Legends to figure

The figure illustrates the response to treatment to daratumumab when given for the first time (Da) and depending on regimens. In patients treated with Da-mono 19.3% had ≥VGPR, in patients treated with Da-IMiDs 56.0% had ≥VGPR, and in patients treated with Da-PI 32.4% had ≥VGPR. Abbreviations: Da-mono=daratumumab monotherapy; Da-IMiD=daratumumab in combination with an immunomodulatory agent; Da-PI=daratumumab in combination with a proteasome inhibitor; ≥VGPR=very good partial response or better; PR=partial response; ≤MR=minimal response or worse

Supplementary figure 4. Response to first daratumumab- containing therapy depending on timing

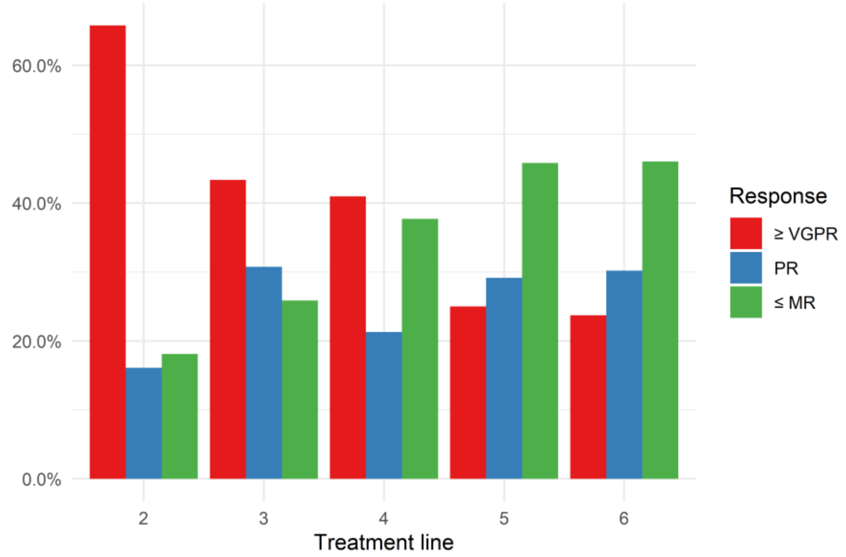

Legends to figure

The figure illustrates the response to treatment with daratumumab when given for the first time and according to timing. Very good partial remission or better was 65.8% in 2. line, 43.4% in 3. line, 41.0% in 4. Line, 25.0% in 5. Line, and 23.7% 6. or later line of daratumumab.

The real-world outcomes of multiple myeloma patients treated with daratumumab as monotherapy, in combination with immunomodulatory drugs and proteasome inhibitors by Szabo et al. Corresponding author AJV.

Supplementary figure 5. Time to next treatment depending on the timing of daratumumab therapy

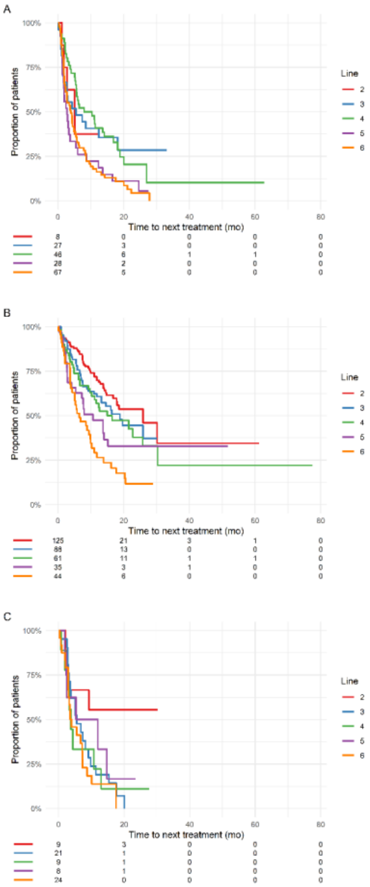

Patients at risk is shown below each figure. A: TNT for Da-mono depending on timing. Cox-regression analysis showed longer TNT in early lines (p=0.002). B: TNT for Da-IMiD depending on timing. Cox-regression analysis showed longer TNT in early lines (p<0.0001). C: TNT for Da-PI. Cox-regression analysis showed a trend for longer TNT in early lines (0.064).

The real-world outcomes of multiple myeloma patients treated with daratumumab as monotherapy, in combination with immunomodulatory drugs and proteasome inhibitors by Szabo et al. Corresponding author AJV.

Supplementary figure 6. Time to next treatment of the first daratumumab therapy depending on cytogenetic abnormalities and timing

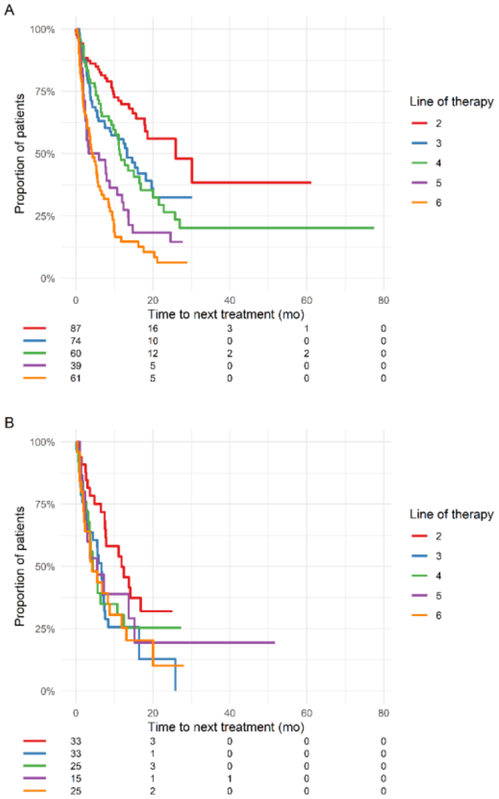

Patients at risk is shown below each figure. High-risk CA was defined as the presence of del17p, t(4:14) or t(14:16). Abbreviations: mo=months . A: TNT for for all combinations of daratumumab-containing line of therapy in patients without high-risk CA depending on timing. Cox-regression analysis showed longer TNT in early lines (p<0.0001). C: TNT for all combinations of Da in patients with high-risk CA depending on timing. Cox-regression analysis showed a trend for longer TNT in early lines (p=0.07).

The real-world outcomes of multiple myeloma patients treated with daratumumab as monotherapy, in combination with immunomodulatory drugs and proteasome inhibitors by Szabo et al. Corresponding author AJV.

Supplementary figure 7. TNT for daratumumab, irrespectively of combination, according to line of treatment compared to treatment not including daratumumab

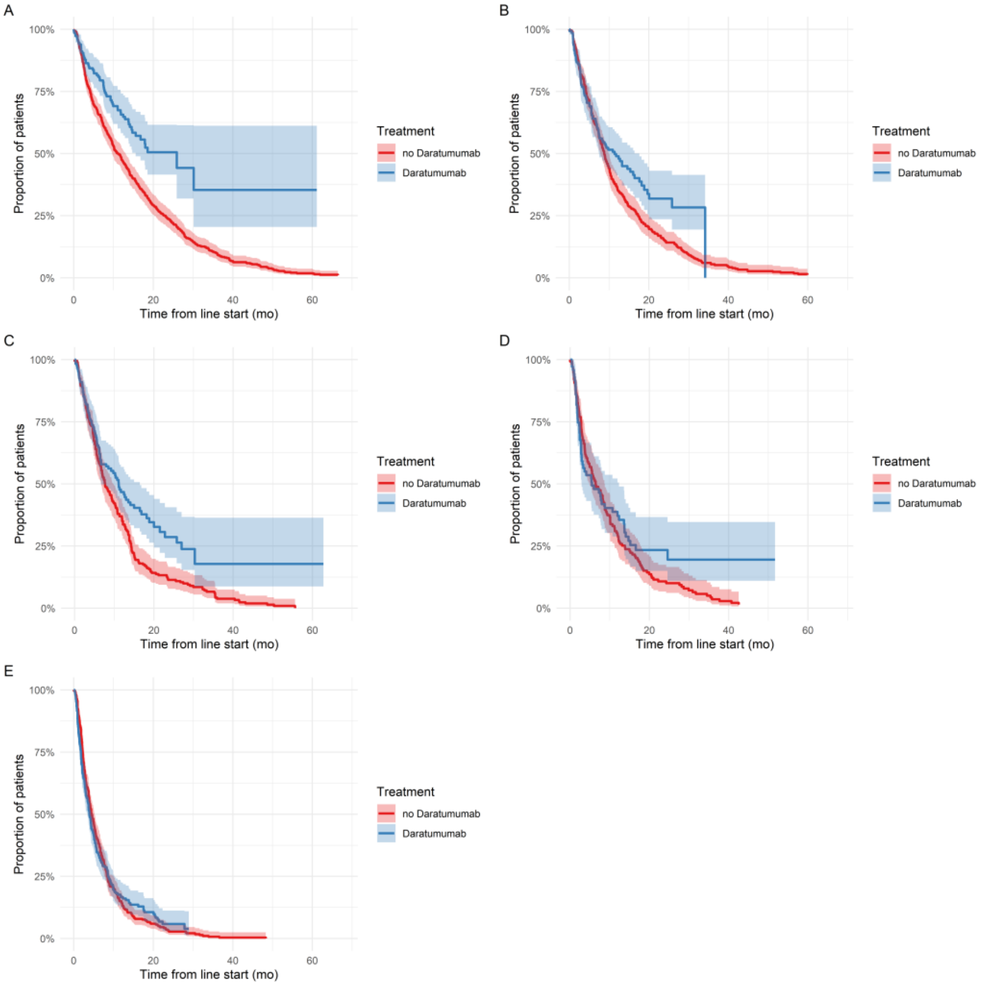

We explored multiple panels of the time to next treatment (TNT) of the second (A), third (B), fourth (C), fifth (D) and sixth or later (E) lines of therapy (LOT). The TNT of the first daratumumab-containing LOT (1Da; blue curve) is compared with the TNT achieved in patients who received the same line of therapy without daratumumab. The TNT for Da was significantly longer in the second ($p<0.0001$), the third ($p=0.012$) and the fourth ($p=0.0005$), but not in the fifth ($p=0.34$) and sixth or later (0.93) LOT compared with the TNT achieved in patients who received the same line of therapy without daratumumab.
