# Supplementary material for: The real-world outcomes of multiple myeloma patients treated with daratumumab
Source: PLoS One. 2021 Oct 13;16(10):e0258487. doi: 10.1371/journal.pone.0258487 (PMC8513840; doi:10.1371/journal.pone.0258487)
Supplement: Attachment — Submitted filename: Rebuttal letter.docx [file pone.0258487.s002.docx]

Dear Francesco Bertolini, MD, PhD

Academic Editor

PLOS ONE

Thank you the constructive reviewer´s comments, and the opportunity to revise our manuscript.

I here send you the revised manuscript. Included is one copy where all changes are highlighted and one clean copy, and below I have commented on the reviewer´s criticism. The changes in response to the reviewers are highlighted in colour.

We thank the reviewers for the suggestions that have improved the manuscript considerably.

*1. Overall survival results should be showed and included. In relapsed/refractory disease, quality of life and survival are the two main objectives. Showing only time to next treatment is a big limitation of the study and the real impact of daratumumab could be biased. A impact beyond PFS and TNT in terms of OS has been found in the original trials and the OS could help to
explore is that is also happening in real world practice. We agree with the reviewer that OS is important. We have inserted OS in Figure 1 and in the text. However, even in real life it requires a long follow up, which will result in data that are uninteresting as we now move into the field of treatment with BiTes and Celmods. Our aim with this publication is to present TNT for daratumumab. Figure 1A show the TNT for the same groups and has not been changed.*

*We have included OS for*

1. *Dara + IMids+ dex*
2. *Dara + Btz + dex*
3. *Dara + dex*
4. *Other*

*from date of start of daratumumab treatment. The data are presented in the text and in figure 1 B. We have calculated the significance in OS between the groups. We have calculated median OS for all patients and inserted in the Table 1 and in in the text.*

*2. The authors are dealing mainly with refractory and relapsed multiple myeloma. That should be stated in the title, the abstract, the methods section and elsewhere. Patients in first line should not be included in this manuscript as it seems not approved in Denmark at that time. Our aim was to include all patient treated with daratumumab nationwide within a given time period and therefore 10 patients (1.6%) treated with daratumumab at first line within clinical trials are included. We believe that it is fair to include these patients and include them in one figure to illustrate that the TNT is longer for patients treated in first line. Inclusion of the 10 patients treated with daratumumab as first line dose not change our conclusions.*

*3. "Timing to treatment" in abstract and results section should be defined. It is related to the number of previous lines of treatment, that would be easier. The other option is the time from diagnosis. But it should be stated more clearly. Timing of treatment is defined as timing of daratumumab in the sequence of all LOT that the patients received throughout the course of their disease. The sentence is inserted in the abstract as well as in the method section.
4. Are patients from clinical trials excluded in this study? This should be clarified in the methods section. 10 patients (1.6%) treated with daratumumab at first line within clinical trials are included. We kindly refer to question no 2.*

*5. Novelty statement should be rewrite, including the number of patients studied and the number of patients with cytogenetic data available. We have included the number of patients in the study and the number of patients with available cytogenetic analysis.*

*6. amp1q results are intriguing. As available with the same technique, the authors should try to include the information about del1p. Also, the power of amp1q plus other abnormalities vs. the amp1q by itself could be also of interest. This is a very interesting question. Unfortunately, analysis of del1p is not routine practice in Denmark and we cannot provide the results. However, we have included the power of amp1q plus other abnormalities vs. the amp1q. The results are inserted in the text and in supplementary Table 6. The following text is inserted at line 227:* but TNT of patient with am1q was longer as compared to patients carrying high-risk CA combined and amp1q (P=0.036; Supplementary Table 9)

| Amp1q | 75 | 9.8 | 6.5-16.9 | 1 |  |
| --- | --- | --- | --- | --- | --- |
| High-risk + amp1q | 55 | 5.6 | 3.6-12.4 | 1.56 (1.03-2.35) | 0.036 |

*7. A chart of the approved treatment during that period of time in Denmark for multiple myeloma in first line and at relapse should be included. A chart has been included as supplementary Table X
8. "Lenalidomide maintenance" (page 6 line 111): it should be described "after ASCT". Thanks for the precision, we have inserted after ASCT in the text as suggested.*

*9. The authors considered at least 10% of cytogenetic abnormalities for prognostication. However, the level is quite in debate in the myeloma community, including even 55% of del17p in some big series. Exploration of other cutoff could be useful, particularly with amp1q or even del17p. We agree with the referee. It is debated in the myeloma community, and many earlier trials do not even include the cut-off of the analysis. Some authors claim that it is irrelevant whether the cut-off is 10 or 60%. The argument is that if the treatment does not work in patients with f. ex. del17p this clone will be the prevailing clone at relapse.*

*10. Page 10, line 185: Da-mono is nor a "combination". Thanks, we have changed the word “combination” to the word “regimen”
11. What regimens were available after 4th LOT for patients with MM vs. daratumumab in monotherapy? (otherwise, 2., 3., 4. system is confused)* The available standard regimens used in the study period are *shown in supplementary table 1 and*  Supplementary table XX, as reported above. The treating physicians could use the standard regimens or off-label combinations on a case-by-case basis.

*We are not sure that we understand the question. Other treatment strategies include doctor´s choice of treatment*

*12. Peripheral neuropathy (16%!) as a toxicity related to daratumumab is quite inconsistent. Authors should describe how many patients developed this complications outside bortezomib treatment. I know that in a registry study is difficult to analyze what was the drug associated with the problem; however, if almost all the cases are in Dara-bortezomib group, it should be stated. Was Bortezomib administered s.c. or i.v.?* In this retrospective analysis toxicities were only registered if they were the cause of discontinuation of a line of therapy. This may have been unclear for the readers, and we therefore added an extra sentence to clarify this in methods and in the legends of Supplementary Table 15.

Supplementary Table 15 is to be viewed in relation to Supplementary Table 14. As shown in Supplementary Table 14, toxicity (in general) was the cause of discontinuation of the first daratumumab-containing line of therapy in 56 cases, which was 13.7% of all discontinuations. Supplementary Table 15 shows the type of toxicity in the 56 cases. Neuropathy was reported as the toxicity leading to discontinuation in 9 cases in total, across all daratumumab regimens, representing 16.1% of discontinuations due to toxicity. In total, this patient number represented 1.4% of the entire cohort of daratumumab-treated patients. The distribution of the 9 cases of neuropathy across the daratumumab regimens is shown in this table: 1 patient in Da-mono, 4 patients in Da-IMiD, 4 patients in Da-PI.

Although this was not registered specifically, all patient in this study were treated with subcutaneous bortezomib, which has been the standard of care in Denmark since 2011.

*13. Table 1 and Table 2 (and even Table 3)are extremely busy. They should be moved to supplementary data and only a summary should b showed in the manuscript. Figures 2B, 2C and 2D could be also moved to supplementary data. We have prepared a summary of the results and moved the full tables as well as Figures 2B, 2C and 2D the supplementary material
14. Figure 3 should be clearer. Combining timing and CA abnormalities should be more to supplementary data. Here, a clear data with the impact of CA according to 2 or 3 groups could be more useful graphically.*

*We understand that the figure may be busy, but we find that it is important to show standard risk as well as TNT for patients with high risk cytogenetic +/- amp1q and amp1q alone. We have removed patients with missing.*

*TNT* *standard*

*TNT for patient with high risk cytogenetic t(4;14); t(14;16); del(17p) and amp1q*

*TNT for patients amp1q*

*TNT for high risk cytogenetic t(4;14); t(14;16); del(17p)*

*We have removed the figures combining timing and CA abnormalities to supplementary data*
