# Supplementary material for: The real-world outcomes of multiple myeloma patients treated with daratumumab
Source: PLoS One. 2021 Oct 13;16(10):e0258487. doi: 10.1371/journal.pone.0258487 (PMC8513840; doi:10.1371/journal.pone.0258487)
Supplement: Attachment — Submitted filename: Rebuttal letter.docx [file pone.0258487.s003.docx]

Dear Francesco Bertolini, MD, PhD

Academic Editor

PLOS ONE

Thank you the reviewer´s comments, and the opportunity to revise our manuscript.

I here send you the revised manuscript. Included is one copy where all changes are highlighted and one clean copy, and below I have commented on the reviewer´s criticism. The changes in response to the reviewers are highlighted in colour.

Reviewer #2: The authors have included many of the suggestions and modified the vast majority of critical points arisen during the first revision of the manuscript. Particularly, the inclusion of overall survival analysis and some methodological modifications. It seems much clearer now for me and I think for the readers too.

I am still worried about the 10 patients included in first line and within a clinical trial. I think even you could mention these patients in the manuscript, they are exceptions of the inclusion criteria in your study and, instead of improving the quality, this information diverts from the main goal of the paper.

We understand the concern from the reviewer´s and we thank you for your comments. Our inclusion criteria were treatment of daratumumab irrespectively of line of therapy. We have inserted a comment about these patients in the results.

Ten patients received daratumumab in combination with lenalidomide in first line as part of the Maia study. The outcome of these patients is presented in figure 2.
